# DORSALIS PEDIS NEUROVASCULAR FLAP, OUR EXPERIENCE

**DOI:** 10.1590/1413-785220233103e267572

**Published:** 2023-09-08

**Authors:** Sérgio Aparecido do Amaral, Bárbara Letícia Ferreira de Carvalho, Antonio Clodoildo Andrade, Maurício Benedito Ferreira Caetano, Luiz Angelo Vieira, Edie Benedito Caetano

**Affiliations:** 1 Pontifícia Universidade Católica de São Paulo (PUC), Faculdade de Ciências Médicas e da Saúde, Sorocaba, SP, Brazil.; 2 Conjunto Hospitalar de Sorocaba, Hand Surgery Service, Sorocaba, SP, Brazil.; 3 Pontifícia Universidade Católica de São Paulo Sorocaba (PUC), Faculdade de Ciências Médicas e da Saúde, Department of Surgery, SP, Brazil.

**Keywords:** Postoperative Complications, Lower Extremity, Upper Extremity, Foot, Surgical Flaps, Complicações Pós-Operatórias, Extremidade Inferior, Extremidade Superior, Pé, Retalhos Cirúrgicos

## Abstract

**Objectives:**

Analyze the donor site morbidity of the dorsalis pedis neurovascular flap in traumatic injuries with hand tissue loss.

**Material and Methods:**

The study involved dorsalis pedis neurovascular flaps that were used to reconstruct the hands of eight male patients, between 1983 and 2003, aged between 21 and 53 years (mean 34.6, SD ± 10.5 years). The size of the lesions ranged from 35 to 78 cm2 (mean 53, SD ± 14.4 cm2). Surgical procedures were performed two to 21 days after the injuries had occurred. The patients were followed up for an average of 10.3 years (ranging 8–14, SD ± 2.1 years).

**Results:**

Regarding the donor site, in one case there was hematoma formation, which was drained; in another case, the skin graft needed to be reassessed. All patients experienced delayed healing, with complete healing from 2 to 12 months after the surgery (mean 4.3, SD ± 3.2 months).

**Conclusion:**

Despite the advantages of the dorsalis pedis neurovascular flap, we consider that the sequelae in the donor site is cosmetically unacceptable. Nowadays, this procedure is only indicated and justified when associated with the second toe transfer. ***Level of Evidence IV; Case series*** .

## RESUMO

**Objetivos:**

Analisar a morbidade da área doadora do retalho neurovascular do dorso do pé em lesões traumáticas com perda de tecido da mão.

**Material e métodos:**

O estudo envolveu retalhos neurovasculares do dorso do pé usados para reconstruir as mãos de oito pacientes do sexo masculino, entre 1983 e 2003, com idades entre 21 e53 anos (média de 34,6, DP ± 10,5 anos). O tamanho das lesões variou de 35 a 78 cm2 (média de 53, DP ± 14,4 cm2). Os procedimentos cirúrgicos foram realizados entre dois a 21 dias após a ocorrência das lesões. Os pacientes foram acompanhados por uma média de10,3 anos (variando de 8 a 14, DP ± 2,1 anos).

**Resultados:**

Quanto ao local doador, em um caso houve formação de hematoma, que foi drenado; em outro caso, o enxerto de pele precisou ser reavaliado. Todos os pacientes apresentaram retardo na cicatrização, com cicatrização completa de 2 a 12 meses após a cirurgia (média de 4,3, DP ± 3,2 meses).

**Conclusão:**

Apesar das vantagens do retalho neurovascular do dorso do pé, consideramos que as sequelas no local doador são cosmeticamente inaceitáveis. Atualmente, esse procedimento só é indicado e justificado quando associado à transferência do segundo dedo do pé. ***Nível de evidência IV; Série de casos*** .

## INTRODUCTION

It is a challenge to properly cover complicated injuries of the extremities, especially hand lesions and whenever structures such as bones, tendons, nerves, and blood vessels are exposed. Studies have described different types of flaps to cover such injuries^1 – 3^ . The dorsalis pedis artery island flaps were originally introduced by McCraw and Fulow^4^ ; Ohmori and Harii^5^ improved the technique using a neurovascular free flap in hand reconstruction for the restoration of hand sensibility^3 – 5^ .

The dorsalis pedis neurovascular flap is a fasciocutaneous flap supplied by branches of the dorsal artery of the foot, which may present anatomical variations^6 – 10^ . In 83% of cases, it originates from the anterior tibial artery^6^ , which may be absent, or it may originate from the fibular artery^6 , 8^ . The venous return is performed by the principal and internal saphenous veins; the innervation is through the superficial and deep fibular nerves^4 , 8^ . Some advantages of this type of flap make it ideal for coverage of hand wounds as it has the potential benefit of being thin and pliable, the anatomical structure is similar to the soft tissue of the hand, its pedicle is fairly long, and its vascular anatomy is reliable. Moreover, it can be easily harvested, with potential to include vascularized structures such as bones and tendons, as well as the superficial and deep peroneal nerves. It also allows restoration of sensibility in the recipient site^7 , 11^ .

However, the use of this flap has been plagued by questions over sequelae in the donor site^12 – 15^ . The aim of this study was to present the results of eight cases in which dorsalis pedis neurovascular flaps were used for the treatment of hand injuries, and to evaluate if this type of flap would be indicated for such cases at the present moment.

## MATERIAL AND METHODS

The study evaluated eight hand reconstruction procedures with dorsalis pedis neurovascular flap transfers between the years 1983 and 2003. The mean age of patients was 34.6 ± 10.5 years, ranging from 21 to 53 years old, and they were all male. The hand reconstruction procedures were carried out in six right hands and two left hands. Most of the hand injuries studied were caused by mechanical trauma. Three were caused by a press machine, three were pinch point injuries, one was a crush injury from a motorcycle accident, and one was caused by electrical burn.

The size of the lesions, measured with a millimeter ruler, ranged from 35 to 78 cm2 (mean of 53 ± 14.4 cm2). None of the patients had loss of bone or tendon tissue; one patient had the index finger amputated, and another patient lost the index, middle, ring, and little fingers. The surgical procedures were performed from 2 to 21 days after the injuries occurred.

**Figure 1:**
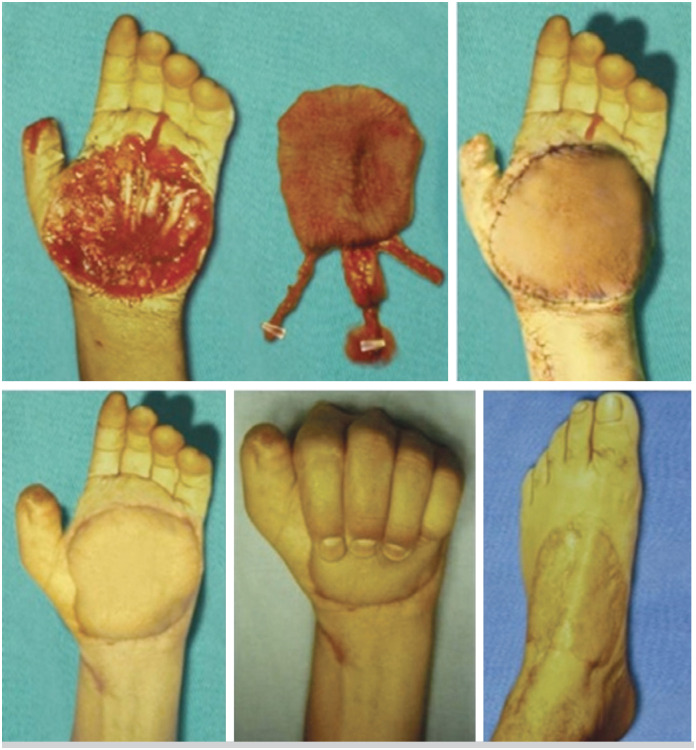
Dorsalis pedis neurovascular flap covering the palm of the hand.

**Figure 2:**
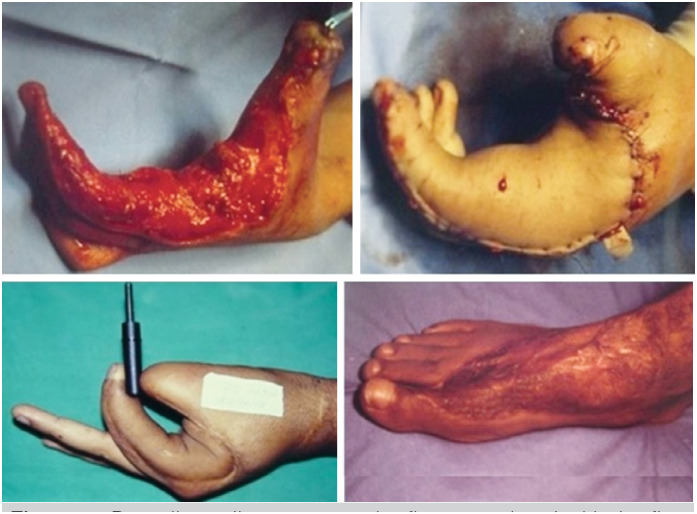
Dorsalis pedis neurovascular flap associated with the first commissure to properly cover the radial side of the hand and middle finger.

**Figure 3:**
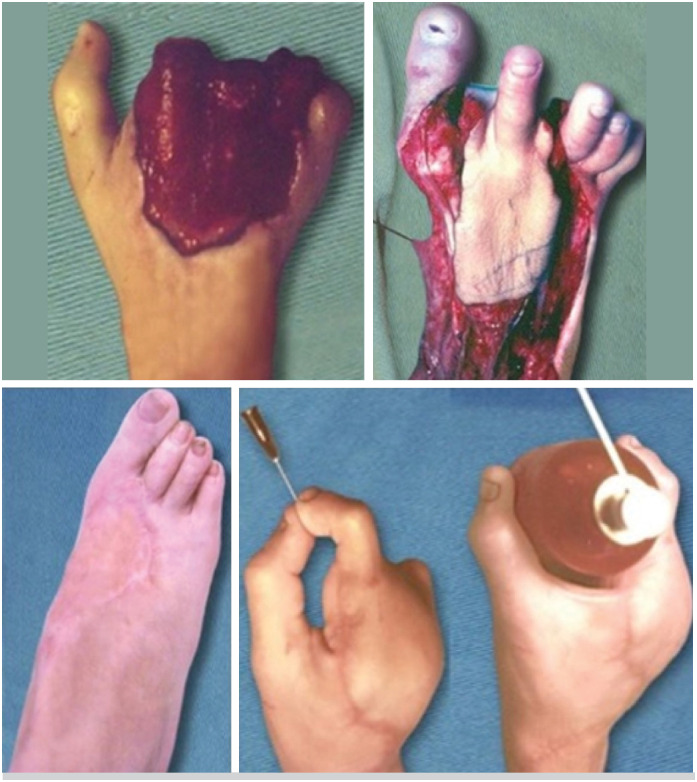
Dorsalis pedis neurovascular flap associated with the transfer of the second toe.

Regarding the recipient sites, three flaps were used to cover the palm of the hand ( Figure 1 ), two to cover the palm and first commissure, one to cover the radial side of the hand and middle finger( Figure 2 ), one was associated with the transfer of the second toe ( Figure 3 ), and one was used to cover a completely degloved hand.

In the preoperative period, the patency of the dorsalis pedis artery was examined by the palpation method or by a Doppler test. With a pneumatic tourniquet between the hallux and the second toe, we proceeded with the distal flap elevation. The mechanical separation of the first and second metatarsal bones enabled the visualization of the first dorsal metatarsal artery to start the dissection. The fascia was included in the flap to avoid separation of the flap artery. The pedicle was only detached from the donor site once the recipient site was adequately prepared. End-to-side anastomosis were performed outside the area of injury. A skin graft was performed immediately after the flap transfer in two limbs, and the other six patients received a graft seven to ten days after the flap transfer. The mean follow up period was10.3 years (8 to 14 ± 2.1 years).

## RESULTS

One patient experienced loss of a small portion of the skin graft by secondary intention healing; another patient had a hematoma that was drained, and all patients had delayed healing beyond 30 days. Healing was completed between 2 and 12 months(mean 4.3 ± 3.2 months).

Restoration of protective sensation was observed in all patients, and all flaps survived. Although no patient had donor or recipient site infections, all of them experienced significant donor site morbidity, especially delayed healing. Table 1 summarizes the data reported in Methods and Results.

**Table 1:** General summary of the informations contained in Methods and Results.

Donor site	Recipient site	Flap size (cm^2^)	Infections	Morbidity	Complications	Healing period(meses) (months)	Sensibility mão	Follow up (years)(anos)
Dorsum of the foot	Palm of the hand	35	N	Y	N	4	Y	10
Dorsum of the foot + hallux	Palm of the hand + middle finger	72	N	Y	N	3	Y	8
Dorsum of the foot	Palm of the hand + 1st commissure	46	N	Y	N	3	Y	10
Dorsum of the foot + 1st commissure	Hand degloving	78	N	Y	Partial lost of the flap	12	Y	12
Dorsum of the foot + 2nd toe	Dorsum qof the hand + digital commissure	50	N	Y	N	3	Y	14
Dorsum of the foot	Palm of the hand	45	N	Y	N	2	Y	9
Dorsum of the foot	Palm of the hand + 1st commissure	48	N	Y	Bruise	5	Y	12
Dorsum of the foot	Palm of the hand	50	N	Y	N	3	Y	8
		53				4,3		10,3
		35				2		8
		78				12		14
		14,4				3,2		2,1

## DISCUSSION

In this study we employed the dorsalis pedis neurovascular flap in 8 patients with the purpose of restoring sensibility in critical areas of the hand. We agree that no other flap described in literature provides comparable results regarding the return of sensibility^11^ . The protective sensation was restored in all 8 patients; however, we deem the donor site morbidity to be unacceptable. We believe that the use of this flap would be justified only in case 4 of this study, where the flap was transferred along with the second toe for reconstruction of the digital commissure.

Some authors have reported that the sequelae in the donor site are not significant^4 , 12 , 15 – 19^ , while other authors consider donor site morbidity to be unacceptable^11 , 12 , 20 – 24^ .

Samson et al.^12^ report that donor site morbidity is significant, and they recommend that this flap should only be used when there are no other options available. To avoid more serious sequelae, the authors recommend that the flap should not be extensive, and the distal edge should be at least 2 cm proximal to the digital commissures.

McCraw and Furlow^4^ report that 11 patients were treated with a dorsalis pedis neurovascular flap, and the donor site morbidity was negligible. Healing was delayed in some patients, but the cosmetic appearance of the donor site was acceptable. They recommend close attention to the donor site trauma to avoid delayed healing. Ohmori et al.^5^ successfully transferred dorsalis pedis neurovascular flaps in five patients. The cosmetic appearance of the donor site was not mentioned in their study.

Krag and Niegels^15^ , report that the dorsalis pedis pedicled island flap was used in 13 patients and the intended purpose was achieved in 12 cases (92.3%). They considered the donor site morbidity to be insignificant.

For Ismail^20^ the resulting sequelae are significant. To avoid damage to the donor site, the flap should have small dimensions that allow primary closure of the donor site. For the author, the need of a skin graft may cause significant damage due to delayed healing.On the other hand, Hallok^21^ considers that direct donor site closure can be problematic, and the damage could be more significant than those resulting from the use of skin grafts.

For Schlenker et al.^17^ , who studied 9 free flaps transfers including 3 dorsalis pedis flaps, the damage to the donor site was not significant.

Daniel and Weilan^23^ report to have performed 18 free flap transfers for hand reconstruction. Two of the cases involved dorsalis pedis neurovascular flaps and there was restoration of sensibility. Healing in the donor site was delayed in both cases; one of the cases had periodic ulceration, and the healing process occurred after 18 months.

Zuker and Manktelow^11^ state that the ideal flap to cover areas must include the superficial and deep peroneal nerves, for sensibility is of utmost importance. For the authors, a careful dissection with preservation of the paratenon of the toe and hallux will minimize donor site damage. They report that the distal edge of the flap must remain at least two centimeters proximal to the digital commissures. Moreover, they observed that the flaps extending to the first commissure did not heal properly.

Caroli et al.^16^ used dorsalis pedis neurovascular flaps in three patients. Extensor tendons were incorporated into the flaps to cover areas with loss of skin and tendons on the dorsum of the hand. They report that there was delayed healing in two of the cases because the flaps were long, and the aesthetic result of the dorsum of the foot was not acceptable.

Vila Rovina et al.^22^ believe that an extensor tendon transfer combined with a dorsalis pedis flap is an excellent technique to repair hand tissue defects. However, they consider the donor site morbidity to be significant and recommend that such technique is only used when there are no other options available. In one of the cases, the authors transferred the second toe with the dorsalis pedis neurovascular flap for reconstruction of the digital commissure.

Wang et al.^24^ used the dorsalis pedis neurovascular flap combined with toe transfer in 15 patients with hand injuries. All flaps survived. At 34.8 months of follow-up, the average subjective satisfaction score was 8. Eleven patients (73.3 %) experienced cold intolerance, dysesthesia, and delayed healing.

Morrison et al.^19^ , performed toe transfers in 44 patients, and dorsalis pedis flap was used in 6 of the procedures. For the authors, the donor site morbidity was not insignificant; delayed healing occurred frequently, but they believe the functional results were beneficial.

Han et al.^18^ treated 25 patients with hand degloving injury using the dorsalis pedis flap associated with the first commissure flap and other flaps. Although they observed ulcer formation and delayed healing in some of the patients, the results were satisfactory.

## Study limitations

Despite the advantages provided by the dorsalis pedis neurovascular flap, we consider that the sequelae in the donor site is cosmetically unacceptable. Nowadays, this procedure is only indicated and justified when associated with the transfer of the second toe, as seen in case 4 of this study.

## CONCLUSION

The flap studied proved to be effective for restoration of protective sensation. As for the cosmetic aspect of the donor site, the results are questionable. We considered the best indication to be a combination of the flap with the transfer of the second toe.
